# Expert consensus on the advantages of traditional Chinese medicine in the treatment of rheumatoid arthritis

**DOI:** 10.1186/s13020-025-01129-7

**Published:** 2025-05-27

**Authors:** Jinping Wang, Xiaoxiao Zhang, Mei Mo, Nan Zhang, Qingwen Tao, Yuan Xu

**Affiliations:** 1 National Center for Integrative Medicine, Department of TCM Rheumatism, China-Japan Friendship Hospital, Beijing, People’s Republic of China; 2 China Association of Chinese Medicine, Beijing, 100029 China; 3 National Key Laboratory of Chinese Medicine Modernization, Tianjin, 300410 China

Rheumatoid arthritis (RA) is a chronic autoimmune disease characterized by progressive erosive arthritis, with a high prevalence and significant disability burden. Despite considerable advancements in RA treatment in recent years, challenges such as limited therapeutic efficacy, drug resistance, severe adverse effects, and the high costs associated with long-term management continue to pose substantial obstacles. These challenges are especially pronounced in patients with early-stage RA, as well as those who experience complications, comorbidities, or severe forms of the disease.

At the "27th Clinical Advantageous Disease Series (Rheumatoid Arthritis) Youth Salon," nearly 20 experts and scholars from the fields of traditional Chinese medicine (TCM), Western medicine, and interdisciplinary studies convened for an in-depth exchange on the clinical needs of contemporary RA management. They also explored the beneficial roles of traditional Chinese medicine in treating RA, emphasizing its strengths at specific stages of the disease. Experts at the symposium recognized TCM's unique advantages in addressing early [1] RA, as well as cases involving low to moderate disease activity, remission, and associated complications or comorbidities [2]. By modulating immune function and restoring immune balance, TCM offers both preventative and therapeutic benefits. Furthermore, integrating traditional Chinese and Western medical approaches provides distinct advantages for managing active disease, refractory cases, and severe complications. This integrated approach can rapidly control disease progression, alleviate symptoms, enhance quality of life, and facilitate rehabilitation [3, 4]. RA is often associated with a range of comorbid conditions [5, 6], further complicating its treatment. TCM offers promising potential in regulating immune responses, alleviating symptoms, and improving the overall constitutional health of patients, which may provide new avenues for the comprehensive management of RA comorbidities [7]. However, there remains a significant gap in high-quality clinical research on the combined use of TCM and Western medicine in treating RA. To address this, the establishment of large-scale clinical cohorts and biological databases is essential, as these resources will underpin the development of precision-targeted therapies and evidence-based clinical treatment protocols [8–10]. In the future, personalized treatment strategies that integrate TCM and Western medicine will likely emerge as a key approach to improving the quality of life for RA patients. The following outlines the expert consensus on the advantages of TCM in RA treatment. A summary of the detailed expert opinions can be found in Fig. 1.

1. Current status and challenges in the treatment of RA

- ➣ Treatment options and issues: The treatment regimens for RA include non-steroidal anti-inflammatory drugs, glucocorticoids, traditional and novel disease-modifying anti-rheumatic drugs (DMARDs). However, these treatments are plagued by issues such as suboptimal efficacy, easy recurrence after discontinuation, long-term drug resistance, and side effects.
- ➣ Treatment difficulties in specific scenarios: Treatment of early-stage RA, patients with comorbidities, co-existing conditions, and severe cases is particularly challenging. Ideal medications are lacking, and the management of comorbidities is complex.

2. Advantages of TCM in the Treatment of RA

- ➣ Preventive and holistic approach: TCM adheres to the principle of "preventing disease before it occurs, preventing deterioration once it occurs, and preventing recurrence after recovery." It effectively reduces RA disease activity, alleviates symptoms, and enhances quality of life.
- ➣ Non-pharmacological therapies: Non-pharmacological therapies, particularly acupuncture, are safe and effective in RA management, with strong evidence supporting their use for pain relief. While therapies like cupping or scraping show benefits in clinical experience, their efficacy requires further validation. These modalities complement conventional care by reducing side effects, improving function, and preventing relapse.
- ➣ Syndrome differentiation and treatment: By differentiating syndromes and adopting personalized treatments tailored to different syndrome types, TCM demonstrates unique advantages, including in the treatment of early-stage RA, low-to-moderate disease activity, remission phases, and management of comorbidities and co-existing conditions.

**Fig. 1:**
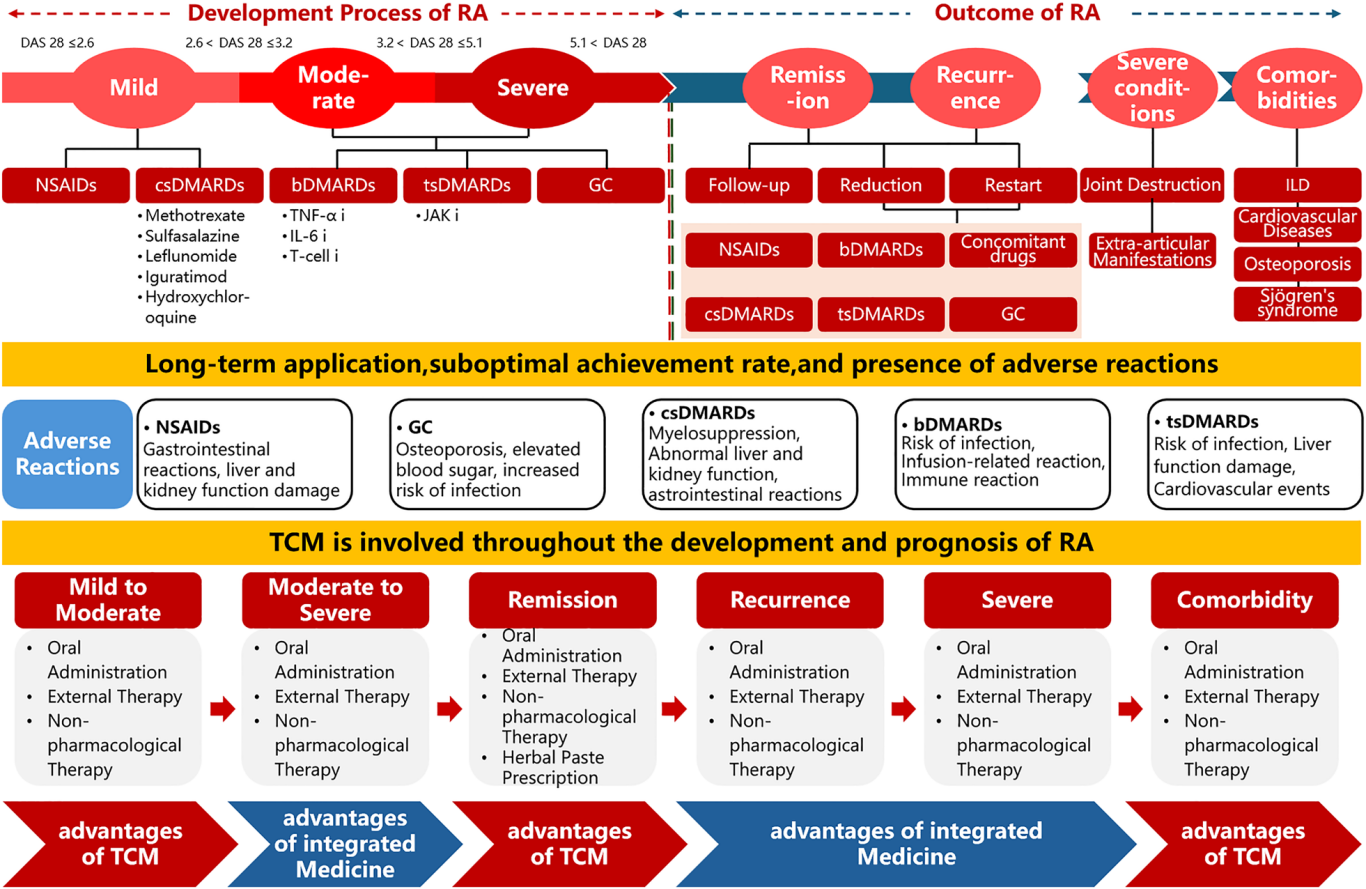
Pathogenesis, progression, and outcome of rheumatoid arthritis, and the therapeutic value of TCM

3. Advantages of Integrated Traditional Chinese and Western Medicine in the Treatment of RA

- ➣ Significant benefits in specific acenarios: The integration of traditional Chinese and western medicine offers significant advantages in RA disease activity phases, refractory RA, and severe complication stages. It rapidly controls disease progression, alleviates patient symptoms, and improves quality of life.
- ➣ Reducing side effects and enhancing efficacy: The combined approach can reduce western medicine side effects, enhance efficacy while reducing toxicity, and facilitate patient recovery.

4. Unique Value of TCM in the Treatment of RA Comorbidities

- ➣ Potential advantages: TCM exhibits potential advantages in regulating immunity, alleviating symptoms, and improving constitution in RA comorbidities, including cardiovascular diseases, respiratory diseases, neuropsychiatric disorders, metabolic diseases, and osteoporosis.
- ➣ Comprehensive treatment approaches: By differentiating syndromes and employing a variety of treatment methods, TCM provides new ideas and means for the comprehensive treatment of RA comorbidities.

5. Future research directions and challenges

- ➣ Lack of high-quality clinical research: High-quality clinical research on the integration of traditional Chinese and western medicine in RA treatment is lacking. There is a need to establish large-sample clinical cohorts and biological databases to identify new biomarkers.
- ➣ Western Medicine Adoption of Chinese Therapies: Western medicine practitioners need to broadly adopt Chinese medical therapies and treatments. Both sides must work together to form consensuses, standards, and guidelines to guide the safe and effective application of traditional medicine in clinical practice.
- ➣ Individualized treatment strategies: In the future, individualized treatment strategies combining traditional Chinese and western medicine will become an important direction for improving the quality of life of RA patients.

In conclusion, TCM presents a valuable and complementary option in the treatment of RA, offering a holistic framework that emphasizes prevention, individualized care, and non-pharmacological therapies. While further research is essential to fully elucidate the underlying mechanisms and refine TCM’s role in RA management, its potential benefits make it an increasingly promising avenue for enhancing the lives of RA patients.

## Data Availability

Not applicable.

## Abbreviations

RA: Rheumatoid arthritis
TCM: Traditional Chinese medicine
